# Adrenal ^131^I-6β-iodomethylnorcholesterol scintigraphy in choosing the side for adrenalectomy in bilateral adrenal tumors with subclinical hypercortisolemia

**DOI:** 10.1007/s00261-015-0452-6

**Published:** 2015-06-04

**Authors:** Lucyna Papierska, Jarosław Ćwikła, Michał Rabijewski, Piotr Glinicki, Maciej Otto, Anna Kasperlik-Załuska

**Affiliations:** Department of Endocrinology, Medical Center of Postgraduate Education, CMKP, ul. Marymoncka 99, 01-809 Warsaw, Poland; Department of Internal Diseases, Metabolism and Endocrinology, Bielański Hospital, Szpital Bielański, ul. Cegłowska 80, 01-809 Warsaw, Poland; Department of Radiology, The Faculty of Medical Sciences, University of Warmia and Mazury, Uniwersytet Warmińsko-Mazurski, ul. Warszawska 30, 10-082 Olsztyn, Poland; Department of Internal Diseases, Diabetology and Endocrinology, Medical University of Warsaw, Mazowiecki Szpital Bródnowski, ul. Kondratowicza 8, 03-242 Warsaw, Poland; Department of General, Vascular and Transplant Surgery, Medical University of Warsaw, CSKUM, ul. Banacha 1a, 02-097 Warsaw, Poland

**Keywords:** Adrenal scintigraphy, Bilateral adrenal tumors, Iodomethylnorcholesterol scintigraphy, Subclinical hypercortisolemia

## Abstract

**Purpose:**

Adrenal scintigraphy with 131I-6β-iodomethylnorcholesterol is considered by several authors the gold standard for assessing tumors with subclinical hypercortisolemia. However, most of the described series consist mainly of cases with unilateral lesions. The aim of our study was to assess whether scintigraphy is useful in choosing the adrenalectomy side in the case of bilateral adrenal tumors with subclinical hypercortisolemia.

**Methods:**

The study focused on 15 consecutive patients with benign bilateral adrenal tumors and subclinical hypercortisolemia. The scintigraphy with 131I-6β-iodomethylnorcholesterol was performed. Fourteen patients underwent unilateral adrenalectomy; the gland with predominant uptake on scintigraphy was removed. Cortisol and ACTH concentrations were measured one and six months after surgery. Post-dexamethasone cortisolemia was assessed six months after surgery. To date, the patients have been under postoperative observation for 1–4 years.

**Results:**

Four patients showed unilateral uptake of radiotracer, and nine patients showed predominant accumulation of radiotracer in one of the adrenal glands. The smaller tumor was predominant in 2 cases. Percentage of activity on the predominant side correlates positively with the difference between tumors’ diameters. Unilateral uptake of radiotracer predicts long-lasting postoperative insufficiency of the second adrenal gland. Excision of predominating tumor led to cessation of hypercortisolemia in all patients.

**Conclusions:**

The corticoadrenal scintigraphy is useful in choosing the side for operation in the case of bilateral adrenal tumors with subclinical hypercortisolemia.

Corticoadrenal scintigraphy (just as with every form of scintigraphy) does not provide a perfect view on morphology of the examined area and fails against other imaging examinations in this respect. However, it should be expected first of all that scintigraphy provides an assessment of the examined tumor’s function after a precise assessment of tumor morphology by X-ray computed tomography (CT) or magnetic resonance imaging (MRI). Scintigraphic imaging of the activity of tumors originating from the adrenal cortex may complement or sometimes even replace catheterization of adrenal veins in the case of aldosteronoma [1], but it seems to be most useful in assessing cortisol-producing adenomas of the adrenal fascicular zone. Scintigraphy was considered the gold standard for assessing adrenal tumors by several authors [2–4]; however, nowadays, it is seldom used in diagnostics of incidentalomas. The cholesterol derivative radiotracer, ^131^I-6β-iodomethylnorcholesterol ([^131^I]-NP-59) accumulates bilaterally in healthy adrenal glands and unilaterally in hormonally active adrenal adenomas or carcinomas [5]. Planar radiographic technique with analysis of the regions of interest (ROI) is the standard method of interpretation. A less frequently employed but more accurate method is using single-photon emission computed tomography (SPECT). SPECT imaging may be useful, in particular, in the diagnostics of ectopic adrenal glands, relapses at the postoperative site or hormonally active metastases, i.e., when an accurate imaging of the radiotracer accumulation site is needed. After completion of a dedicated CT or MRI of adrenal region, if there is still need to confirm tumor hormonal activity, radionuclide scintigraphy will be sufficient imaging modality [2, 5, 6].

Bilateral tumors constitute about 15% of incidentally detected adrenal tumors [2, 7, 8]. They may cause overt clinical or subclinical hypercortisolemia. Some authors state that they have found a higher incidence of subclinical Cushing’s syndrome among patients with benign phenotype bilateral adrenal tumors compared to patients with unilateral lesions [2, 8, 9].

Correct approval for surgical treatment is particularly difficult in case of bilateral lesions. There can only be hormonal activity in one of the tumors; thus, it pays to work out a method for unmistakably identifying the hormonally active tumor that enables a unilateral instead of bilateral adrenalectomy to be performed. Catheterization of adrenal veins with determination of the concentration gradient between them would be the best method in such cases [10, 11]. However, it is an invasive procedure, and patients with hypercortisolemia are more at risk of complications, i.e., thrombosis in the catheterized vessel or damage of the vessel, than others [12]. In the case of bilateral lesions, it is most often assumed that the larger tumor is the hormonally active one; however, the dimensions of tumors differ slightly in some patients. For assessment of incidentalomas, some researchers have carried out radio-labeled norcholesterol scintigraphies that correlated well with adrenal cortex function [2–4], but in the series described in their papers most of the tumors (85–95%) were unilateral lesions.

The aim of our study was to assess whether scintigraphy is useful in choosing the adrenalectomy side in the case of incidentally detected bilateral adrenal tumors with subclinical hypercortisolemia.

## Materials and methods

The material consisted of 15 patients with incidentally discovered bilateral adrenal tumors and subclinical hypercortisolemia. CT image phenotypes definitely corresponded to benign lesions in all the subjects: the values of attenuation on unenhanced scans were <10 HU (−19 to +8 HU). None of the patients had evident somatic features of hypercortisolemia, however, 13 patients had hypertension, 5 had diabetes mellitus, and 10 had hyperlipidemia. The adopted criterion for the diagnosis of subclinical hypercortisolemia was a cortisolemia of >3 mcg/dl after a dexamethasone suppression test and ACTH concentration of <10 pg/ml (according to criteria proposed by Eller Vanicher and coauthors [13]). Subclinical hyperaldosteronism was ruled out by a calculation of the PAC/PRA ratio and congenital adrenocortical hyperplasia by a determination of the 17-OH-progesterone (17-OHP) concentration. Considering the possibility of mixed tumors of the adrenal cortex and medulla, all patients underwent a determination of 24-h urinary excretion of methoxycatecholamines, which ruled out subclinical catecholamine hypersecretion. Scintigraphy was performed 72 h after intravenous administration of the radiotracer, and, in the case of weak accumulation of the radiotracer, an additional time after 144 h. No patients with subclinical hypercortisolemia underwent initial suppression with dexamethasone before scintigraphy. Thyroid iodine uptake was blocked by oral administration of Lugol’s solution for 2 days. This examination was assessed with a semi-quantitative method. The uptake patterns were classified into four types:

- type 1—no radiotracer uptake in the adrenal glands;
- type 2—unilateral uptake without accumulation on the contralateral side (100/0%).
- type 3—bilateral uptake with distinctly predominant one side (>66% of total activity): “predominant” = 66–75% of total activity on the predominant side (70% was assumed for calculations); “markedly predominant” = >75% of total activity on the predominant side (85% was assumed for calculations);
- type 4—bilateral symmetric uptake (L = R) (radiotracer uptake of 50% in each tumor was assumed for calculations).

To evaluate the correlation between percent of uptake and tumor diameters/hormone concentrations we used Pearson’s analysis of correlation. Values were considered statistically significant at *P* < 0.05. The calculations were performed by STATISTICA™.

Fourteen patients in the tested group underwent unilateral laparoscopic adrenalectomy. The adrenal gland with predominant uptake on scintigraphy was operated, irrespectively whether it harbored the larger tumor or not. In cases with symmetric uptake, the larger tumor was operated. Hydrocortisone was administered parenterally at a dose of 150–200 mg/day on the day of surgery and on day 1 and day 2, and then the treatment was continued using an oral 30-mg/day administered in two divided doses. The dose was tapered down individually according to patient’s clinical state (usually by 5 mg every 1–2 weeks) at ambulatory visits.

During the second month after surgery, 24 h after the last dose of hydrocortisone, a determination of cortisol and ACTH concentration was carried out at 08^00^ h and of cortisol at 22^00^ h, and then these concentrations were determined half a year after surgery and every year from then on. The dexamethasone suppression test with cortisol concentration measurement was performed half a year after surgery, then every year thereafter. The patients have been under postoperative observation for one (two patients) to 4 years (7 patients), none of them had the recurrence of hypercortisolemia.

## Results

**Fig. 1:**
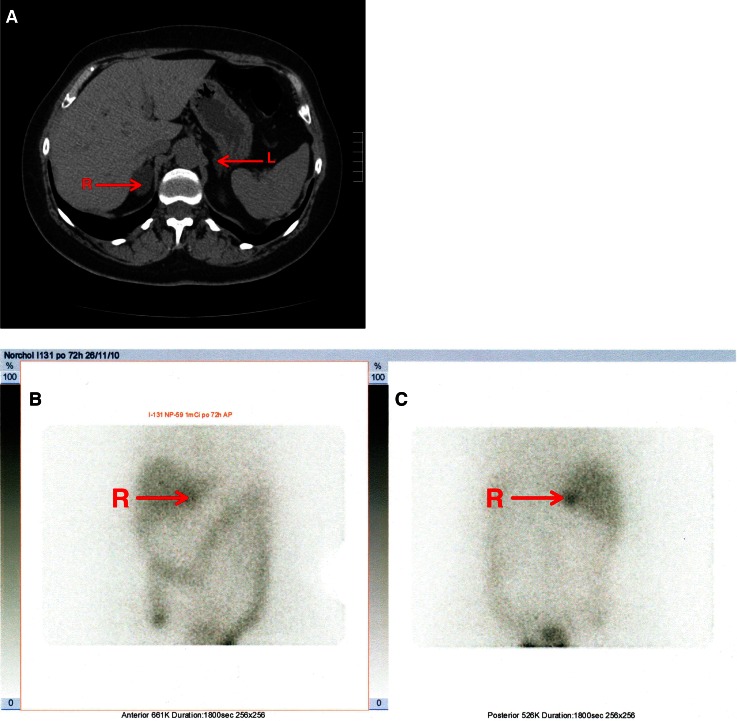
Unilateral uptake of the tracer in the right adrenal gland (with the bigger tumor). **a** CT (the arrows point to the both tumors); **b** anterior planar view of scintigraphic study; **c** posterior planar view.

**Fig. 2:**
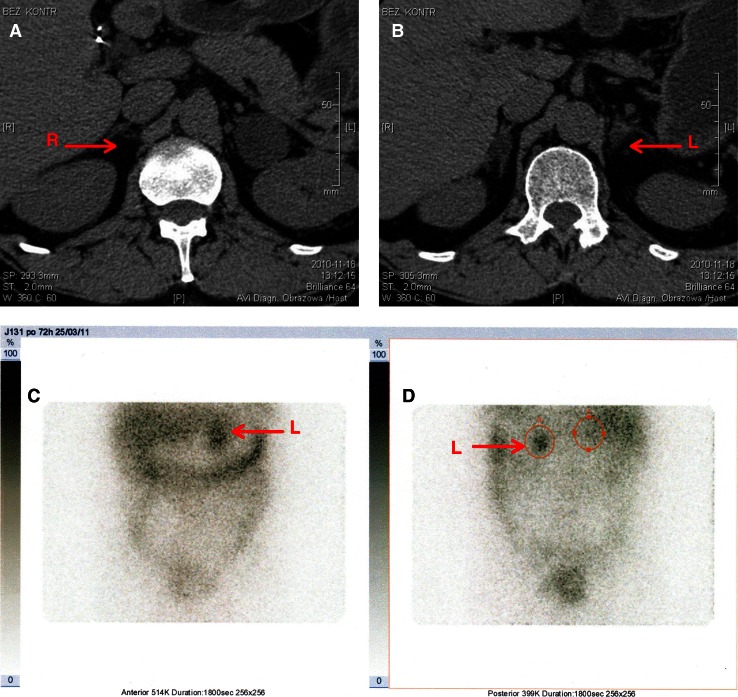
Predominant radiotracer accumulation (85%) in the left adrenal gland (with the bigger tumor). **a**, **b** CT (the arrows point to the both tumors); **c** anterior planar view of scintigraphic study; **d** posterior planar view.

**Fig. 3:**
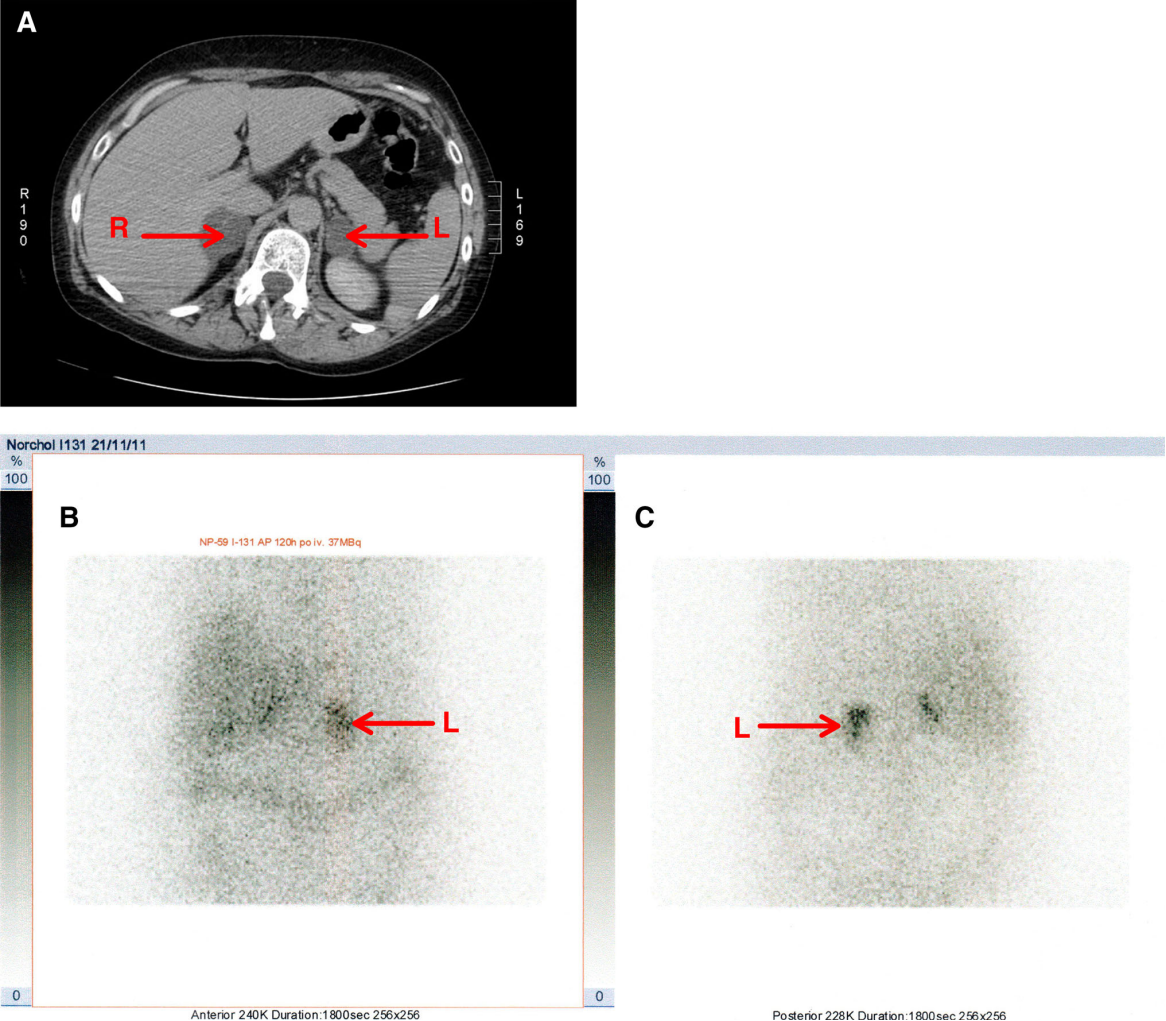
Predominant uptake in the left, smaller tumor with trace accumulation in the right (harbouring the bigger lesion) adrenal gland. **a** CT (the arrows point to the both tumors); **b** anterior planar view of scintigraphic study; **c** posterior planar view.

Four patients (27%) showed only unilateral uptake with no radiotracer accumulation in the contralateral adrenal gland 72 h after the administration of radiotracer (Fig. 1). Predominant accumulation (>66% of total activity) was demonstrated in one of the adrenal glands in nine patients (60%) (Fig. 2). The uptake was symmetric in two patients (13%). The larger tumor was the predominant one on scintigraphy in 11 cases, but predominant uptake was present in the smaller tumor with trace accumulation on the contralateral side in two patients. A smaller by 1-cm tumor was predominant in one case and smaller by 2.3 cm tumor in another case (Fig. 3).

**Fig. 4:**
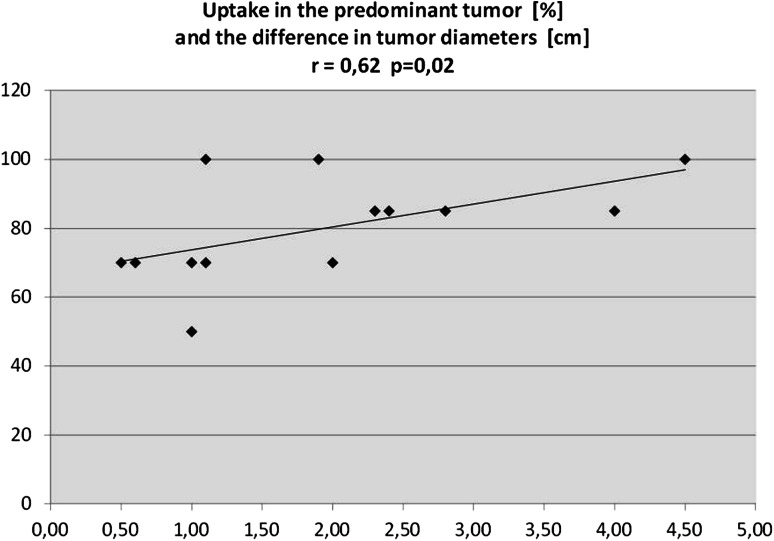
Percentage of activity on the predominant side correlates positively with the difference between diameters of the tumors.

From an analysis of correlations it appeared that the percentage of activity on the predominant side correlates positively but weakly with the difference between tumors’ diameters (*r* = 0.43; *P* = 0.06). After the exclusion from the analysis of two patients with predominant uptake in the smaller tumor, the correlation became stronger (*r* = 0.62; *P* = 0.02) (Fig. 4). The percentage of activity did not correlate with tumor size nor cortisol nor ACTH concentrations.

### Uptake in scintigraphy and postoperative function of the adrenal gland

*Unilateral uptake* of radio-labeled norcholesterol with complete uptake inhibition in the contralateral adrenal gland was found in four patients (27%). The uptake was present in the larger (by 1.2–4.5 cm) tumor in all the cases. The cortisol concentration after the dexamethasone suppression test was on average 6.15 ± 1.2 mcg/dl in these patients and did not differ significantly from that in the other patients. One of these patients refused surgery. Half a year after surgery, three patients with unilateral uptake still presented with postoperative insufficiency of the other adrenal gland, whereas the other patients who were operated on already had normal adrenal function. A secondary hypofunction of the remaining adrenal gland was diagnosed based on persisting inhibition of ACTH (<10 pg/ml), low concentrations of cortisol (<10 mcg/dl in the morning), low 24-h urinary excretion of cortisol (<25 mcg/day), and 17-hydroxy-corticosteroids (<2.2 mg/day) and clinical symptoms (muscular pains, nausea, hypotension during an attempt to withdraw hydrocortisone). Normal concentrations of ACTH and cortisol were found only 2 years after surgery in these patients.

*Predominant uptake on one side* with a slight uptake in the contralateral adrenal gland was detected in nine patients (60%). Predominant uptake in the larger (by at least 1 cm) tumor (1.1–3 cm) occurred in five patients, whereas in two patients predominant uptake was present in the smaller (by 1 and 2.3 cm) tumor. In two persons, predominant uptake was detected in the tumor that was only slightly larger in diameter (a difference of 0.5 and 0.6 cm, respectively). The concentrations of cortisol were low (<10 mcg/dl), and the concentrations of ACTH still remained below the lower normal range (i.e., below 10 pg/ml) in all patients during the second month after surgery. Therefore, a strategy consisting of a gradual hydrocortisone dose decrease was employed. None of the patients had a secondary adrenocortical insufficiency after the withdrawal of hydrocortisone half a year after surgery.

*Symmetric bilateral uptake* was found in two patients (13%) in whom the difference between tumors’ diameters was 0.3 and 1 cm. The larger tumor was operated on first. These patients had no secondary adrenal insufficiency after the adrenalectomy, In one of them, after 6 months, the ACTH concentration was low, and the cortisol concentration was too high (3.6 mcg/dl) after dexamethasone suppression test. This patient gained weight of 5 kg and, as before surgery, has treatment-resistant arterial hypertension. Repeated scintigraphy during the administration of dexamethasone showed radiotracer uptake in the non-operated left adrenal gland. As histology revealed a nodular hyperplasia in the excised right adrenal gland, the surgeon decided to perform a total adrenalectomy. A reduction in arterial pressure values and a continuous loss of body mass (12 kg in total during 2 years) was achieved after the second surgical procedure; this patient receives at present 15–20 mg of hydrocortisone daily.

Proper cortisol concentrations after dexamethasone suppression were found after the consecutive 1 and 2 years in all patients.

The individual results for each patient are shown in Table 1.

**Table 1:** Scintigraphy, preoperative and postoperative hormonal results

	Gender/age	Tumor diameter (cm)		Radiotracer uptake	Liberated side	Preoperative			Postoperative (2™^1^ month)		Postoperative (after 6 months)		
		R	L			Morning cortisolemia (mcg/dl)	Morning ACTH (pg/ml)	Cortisolemia after 1 mg DEXA (mcg/dl)	Morning cortisolemia (mcg/dl)	Morning ACTH (pg/m)	Morning cortisolemia (mcg/dl)	Morning ACTH (pgM)	Cortisolemia after 1 mg DEXA (mcg/dl)
1	F,58	3.0	7.5	L	L	17.1	1	7.2	5.7	2	7	3	Not done
2	F,63	4.3	1.5	R » L	R	13.4	5	4.88	8.1	6	11.2	17	1.8
3	F,67	2.2	2.8	R < L	L	13.6	9	4.6	9	8	12.8	24	1.9
4	F,56	3.3	1.4	R	R	18.8	2	6.34	8.2	4	13.7	4	1.2
5	F,52	3.0	4.0	R = L	L	16	7	11.2	16.2	6	14.2	3	3.6
6	F,58	4.5	1.8	R	R	10.2	5	4.4	4.8	2	8	9	1.3
**7**	**F.59**	**4.1**	**1.8**	**R** **«** **L**	**L**	**17.9**	**9**	**3.7**	**9.6**	**4**	**14.3**	**2″**	**1.2**
8	F,59	2.4	4.4	R < L	L	25	9	4.2	9.1	7	13	15.7	1.97
9	F, 59	3.0	5.4	R « L	L	12	2	3.7	7.8	4	16.2	24.4	1.5
10	F,58	2.9	3.2	R = L	L	18.6	4	4.9	12	6	17.1	16	2.9
**11**	**F.59**	**1.4**	**2.4**	**R** **>** **L**	**R**	**21**	**6**	**7.7**	**7.3**	**5**	**12.6**	**19.6**	**1.2**
12	F,60	4.1	3.5	R > L	R	23	2	8.8	4	2	13,8	22,1	<1
13	F,54	4.2	3.1	R	**–**	17.3	5	6.6	Non-operated				
14	F,66	2.1	3.2	R < L	L	19.8	5	4.8	6.1	2	8.9	13	<1
15	M,53	1.8	5.8	R « L	L	12.2	4	3.4	7.2	6	11.9	36	<1

In patients 7 and 11 (bold) the smaller tumor was predominant on scintigraphy

## Discussion

Correct clinical evaluation and exact analysis of hormonal tests carried out in the patients before surgery warrant effectiveness of the operation. In considering the criteria for approval of surgical treatment in patients with subclinical hypercortisolemia, Eler-Vainicher emphasizes that postoperative secondary insufficiency of the other adrenal gland is definitive proof of tumor hormonal activity [14]. The majority of papers regarding the indication to adrenalectomy discuss the hormonal criteria which must be fulfilled to make a decision about surgery. At least two of the following abnormal results should be found: a cortisol concentration of >3 mcg/dl after dexamethasone, an ACTH concentration of <10 pg/ml, a cortisol concentration of >4 mcg/dl at midnight, and 24-h urinary excretion of free cortisol of >70 mcg [13, 15–17]. In our study, we decided to use the first two hormonal criteria as the simplest ones with respect to execution and repetition, as well as in ambulatory settings. However, even an unquestionable diagnosis of hypercortisomia in the case of bilateral tumors did not ensure successful surgery because the lateralization of secretory function still remained unclear.

Accumulation of radiolabelled norcholesterol in the adrenal glands is evidence of adrenocortical hormonal activity. Therefore, we considered scintigraphy to be potentially valuable for choosing the adrenal gland for surgery in the case of bilateral lesions and subclinical hypercortisolemia. Scintigraphy was considered to be the gold standard for assessing incidentalomas by several authors [2–4]; however, in the series described in their papers, most of the tumors (85–95%) were unilateral lesions. A unilateral radiotracer uptake was found in all the cases of cortisol concentration of >5 mcg/dl after dexamethasone suppression test and such uptake predicted insufficiency of the second adrenal gland after surgery [2, 3]. In our study, a postoperative insufficiency of the second adrenal gland lasting two years occurred in 3 of 4 patients with only unilateral uptake (the fourth person refused surgery), and the preoperative cortisol concentration after dexamethasone suppression test was above 5 mcg/dl in three of these patients. Furthermore, postoperative decrease in cortisolemia did not occur in the two cases of bilateral uptake, and a deteriorated clinical state (increment of body mass, poor control of arterial hypertension), an abnormal dexamethasone suppression test, and a low concentration of ACTH 6 months after surgery were the indication for the second adrenalectomy in one of these patients. On the other hand, the other patient with symmetric radiotracer uptake in whom hydrocortisone was already discontinued during the second month after surgery did not require repeated surgery. Her post-dexamethasone cortisol concentration was borderline (2.6 mcg/dl), and neither an inhibition of ACTH release nor deteriorated general condition was observed in this patient. The above finding is consistent with the results of several studies in which an improvement of the clinical state following the removal of one of the tumors was observed even in the presence of symmetric uptake on scintigraphy without postoperative insufficiency of the other adrenal gland; thus, the hormonal function was certainly bilateral before surgery [18, 19].

In the group described by Iacobone that consisted of seven patients with differences between tumors’ diameters of >2 cm who underwent resection of the adrenal gland harboring the larger tumor and showing predominant uptake of labeled norcholesterol, a marked clinical improvement was achieved in six patients, including two patients with permanent secondary insufficiency of the other adrenal gland [18]. The surgical treatment was not successful in case of similar dimensions of enlarged adrenals, which was accompanied with symmetric uptake of NP-59. The correlation between the percentage of radiotracer uptake by the predominant adrenal gland on scintigraphy and the difference between tumors’ diameters discovered by us also supports the thesis that the greater the difference between lesions’ dimensions, the greater the difference in secretory activity. However, in individual cases the bigger size was not always evidence of tumor secretory activity: in two patients smaller tumors were predominant on scintigraphy (difference between diameters of 2.3 and 1 cm), and in two other patients the only slightly larger tumor was predominant (difference 0.6 and 0.5 cm). The literature regarding bilateral adrenal tumors indicated that tumors larger by at least 1 cm should be operated on when unilateral adrenalectomy is performed [18–20]; thus, the non-secreting side would be operated in the two first persons. If the difference between diameters is less than 1 cm, a selective bilateral excision of fragments of adrenal glands harboring focal lesions is often performed [21–23]; however, execution of such a surgical procedure in two patients would be unnecessary. In addition, histopathological examination showed a macronodular hyperplasia in these female patients; thus, they were at high risk of new focal lesions in the remaining fragments of adrenal glands.

## Conclusion

Corticoadrenal scintigraphy is useful in choosing the operation side in the case of bilateral adrenal tumors with subclinical hypercortisolemia. Unilateral radiotracer uptake on scintigraphy predicts a long-lasting insufficiency of the other adrenal gland after surgery.
